# Epidemiology of Tuberculosis in a High HIV Prevalence Population Provided with Enhanced Diagnosis of Symptomatic Disease 

**DOI:** 10.1371/journal.pmed.0040022

**Published:** 2007-01-02

**Authors:** Elizabeth L Corbett, Tsitsi Bandason, Yin Bun Cheung, Shungu Munyati, Peter Godfrey-Faussett, Richard Hayes, Gavin Churchyard, Anthony Butterworth, Peter Mason

**Affiliations:** 1 London School of Hygiene and Tropical Medicine, London, United Kingdom; 2 Biomedical Research and Training Institute, Harare, Zimbabwe; 3 National Institute of Health Research, Harare, Zimbabwe; 4 Department of Medical Laboratory Sciences, University of Zimbabwe, Harare, Zimbabwe; Stop TB-World Health Organization in Switzerland

## Abstract

**Background:**

Directly observed treatment short course (DOTS), the global control strategy aimed at controlling tuberculosis (TB) transmission through prompt diagnosis of symptomatic smear-positive disease, has failed to prevent rising tuberculosis incidence rates in Africa brought about by the HIV epidemic. However, rising incidence does not necessarily imply failure to control tuberculosis transmission, which is primarily driven by prevalent infectious disease. We investigated the epidemiology of prevalent and incident TB in a high HIV prevalence population provided with enhanced primary health care.

**Methods and Findings:**

Twenty-two businesses in Harare, Zimbabwe, were provided with free smear- and culture-based investigation of TB symptoms through occupational clinics. Anonymised HIV tests were requested from all employees. After 2 y of follow-up for incident TB, a culture-based survey for undiagnosed prevalent TB was conducted. A total of 6,440 of 7,478 eligible employees participated. HIV prevalence was 19%. For HIV-positive and -negative participants, the incidence of culture-positive tuberculosis was 25.3 and 1.3 per 1,000 person-years, respectively (adjusted incidence rate ratio = 18.8; 95% confidence interval [CI] = 10.3 to 34.5: population attributable fraction = 78%), and point prevalence after 2 y was 5.7 and 2.6 per 1,000 population (adjusted odds ratio = 1.7; 95% CI = 0.5 to 6.8: population attributable fraction = 14%). Most patients with prevalent culture-positive TB had subclinical disease when first detected.

**Conclusions:**

Strategies based on prompt investigation of TB symptoms, such as DOTS, may be an effective way of controlling prevalent TB in high HIV prevalence populations. This may translate into effective control of TB transmission despite high TB incidence rates and a period of subclinical infectiousness in some patients.

## Abstract 2

When occupational clinics provide free smear and culture-based investigation of tuberculosis symptoms, it can lead to prompt investigation and may aid TB control in high HIV-prevalence populations.

## Editors' Summary

**Background.:**

Around eight million people develop tuberculosis (TB) disease every year and of these nearly two million die. However, many more people are infected than have symptoms; perhaps one-third of the world's population is currently infected with TB. Most people infected with TB have what is termed “latent infection,” or in other words they are infected with the bacterium but do not experience any symptoms of disease. Individuals infected with TB who also have a weakened immune system, for example through HIV/AIDS, are much more likely to develop TB disease. In some regions HIV is very common—for example, approximately 11% of sub-Saharan African adults are HIV positive—and because of this cases of TB disease have risen substantially as HIV spreads. The Word Health Organization has a recommended international strategy for control of TB called “DOTS” (Directly Observed Therapy, Shortcourse). Among the five main elements of DOTS are mechanisms for promptly diagnosing and treating people who have TB disease. It is hoped that this strategy will help to reduce the number of new cases of TB diagnosed each year, because individuals promptly diagnosed and treated will then be less likely to transmit the disease to others.

**Why Was This Study Done?:**

In this study the investigators wanted to find out if intensive DOTS, combined with giving people better access to test facilities to diagnose TB disease, could be effective in reducing the spread of TB from one person to another in Africa. It is not clear whether DOTS alone can control the spread of TB in populations with high numbers of HIV-positive people already infected with TB and so at high risk of going on to develop TB disease. Specifically, they wanted to collect data on the number of new TB cases being diagnosed per year and how that related to the proportion of the overall population that had infectious undiagnosed TB at any given point in time. They also wanted to find out whether providing good access to services for diagnosis and treatment of TB would affect either the number of new TB cases or the proportion of a given population that had infectious undiagnosed TB.

**What Did the Researchers Do and Find?:**

This research study was carried out as part of a trial in which two different strategies for providing testing and counseling for HIV in the workplace were being compared. The trial took place within 22 companies in Harare, Zimbabwe, where HIV is very common in the adult population. Along with HIV testing and counseling, the trial provided for close follow-up and testing of anyone presenting with TB-like symptoms, with the aim of detecting as many cases in the population as possible. At the end of the two-year period, all workers were checked for undiagnosed TB disease, and cultures were carried out to find out how many of these people had infectious TB (but who might not necessarily have had symptoms). 6,440 workers were recruited into the study, of whom 19% were HIV positive. During the period of follow-up, 106 cases of TB were seen, and HIV-positive workers were far more likely than HIV-negative workers to experience TB disease. At the end of the study, 4,668 workers were checked for the presence of undiagnosed TB and 27 individuals were found to be affected, but not all of these people experienced any symptoms of disease.

**What Do These Findings Mean?:**

At the end of this study, the proportion of workers found to have undiagnosed TB was fairly low—lower than the level found in other studies carried out in other parts of the world with a high burden of TB disease but low burden of HIV. The researchers therefore concluded that the systems set up within the trial (for close follow-up and testing for TB disease) were an effective way of controlling the overall proportion with infectious TB, even though HIV infection rates were also high. This is likely to mean that the spread of TB infection to others—a prerequisite for achieving TB disease control—was also well controlled. However, more intensive efforts to reduce the risk of TB disease in HIV-positive Africans already infected with TB are also needed, although this study did not aim to find out about the impact of such strategies.

**Additional Information.:**

Please access these Web sites via the online version of this summary at http://dx.doi.org/10.1371/journal.pmed.0040022.

- Information from the World Health Organization on DOTS, the internationally recommended TB control strategy; a factsheet on TB is also available
- The STOP TB Partnership is an international initiative involving several agencies seeking to combat the rise of tuberculosis
- The US National Institute of Allergy and Infectious Diseases also publishes a factsheet for patients
- US Centers for Disease Control, information for patients and professionals about TB

**Box 1: Definitions of Prevalent and Incident TB as Used in This Article**

Point prevalence of TB: the fraction of people surveyed at any given point in time with active TB (here given as the number of cases per 1,000 population). In this study, patients on TB treatment were not considered to have active TB unless they were culture positive.

Incidence of TB: the rate at which TB develops over time (here given as the number of cases per 1,000 person-years).

Determining incidence requires longitudinal (cohort) studies, whereas cross-sectional (prevalence) surveys are used to determine prevalence.

## Introduction

Tuberculosis (TB) disease can result from either rapidly progressive disease following recent infection with Mycobacterium tuberculosis or from reactivation of latent TB infection. Reactivational disease predominates in countries that have achieved good control of transmission, but most disease in endemic countries is due to recently transmitted infection [1]. Accordingly, directly observed treatment short course (DOTS), the TB control strategy of the World Health Organization (WHO), aims to reduce the burden of prevalent smear-positive TB through prompt diagnosis and effective treatment of symptomatic patients with infectious disease [1]. In theory, successfully reducing point prevalence will lead to falling TB incidence rates as TB transmission goes into decline (see Box 1) [1]. DOTS has had notable success in a number of countries with low HIV prevalence, and world-wide, it is considered one of the most cost effective of all health interventions [2].

Trends are very different in Africa, however, with the HIV epidemic driving rapid increases in TB case-notification rates despite implementation of DOTS [3,4]. A pressing and undetermined question is whether or not DOTS has also failed to contain TB transmission rates [4,5]. This cannot be assumed, as TB incidence would be expected to rise during the course of an HIV epidemic, even if TB transmission rates were in decline, simply because of increased numbers of highly susceptible individuals [4].

Prevalent infectious TB is the direct source of TB transmission events (Box 1). Evidence from South African gold miners suggests that a DOTS-based approach successfully controlled prevalent TB during a severe epidemic of HIV and HIV-related incident TB [5]. These divergent trends between prevalence and incidence were ascribed to much more rapid self-presentation of HIV-related TB, and offer hope that TB transmission rates were also contained [5]. However, gold miners are unusual because of occupational exposure to silica dust, and although the point prevalence of TB was stable, it was high in both HIV-positive and HIV-negative subpopulations [5]. Also, studies among HIV-positive persons attending for voluntary counselling and testing and in home-based HIV/AIDS care patients have consistently reported high rates of undiagnosed prevalent TB [6–8].

The main aims of this study were to investigate the impact of HIV on the point prevalence and incidence of TB, and to investigate the extent to which infectious (culture-positive) prevalent TB can be controlled by ready access to diagnosis and treatment of symptomatic TB disease in a high HIV prevalence population. For logistical and ethical reasons, the study was nested within a cluster-randomised trial comparing two different strategies of providing voluntary counselling and HIV testing (VCT) at 22 different workplaces in Harare, Zimbabwe. Company clinics were the unit of randomisation, and both VCT strategies were linked to the same package of basic HIV care, including isoniazid preventive therapy for HIV-positive, tuberculin skin test (TST)-positive employees [9].

## Methods

### Study Participants and Cohort Follow-Up

Twenty-two small and medium-sized enterprises (100 to 600 employees) in Harare were enrolled between September 2001 and July 2002, and followed up for 2 y at each site. Most were manufacturers. None involved exposure to silica dust or other occupational risk factors for TB, and none provided accommodation. All employees were asked to consent to a baseline questionnaire including questions about smoking, previous TB treatment, and household contact, and to provide blood for HIV testing, with written informed consent. There was no active screening for TB symptoms or disease at enrolment, but employees were informed that diagnosis and management of common adult illnesses, including HIV and TB, could be obtained through their occupational clinic. VCT was available for workers wishing to know their HIV status. Companies were randomly allocated to either company clinic or voucher-based VCT in a cluster-randomised study [9]. Payrolls were checked every 3 mo for loss to employment and new employees.

Company clinics were provided with a project nurse trained in integrated management of common adult illnesses, adapted from WHO guidelines [10]. Patients presenting with cough for 3 wk or more were investigated with three sputum smears and cultures, including one early morning specimen (“spot-morning-spot”), followed by repeat specimens and chest radiography if symptoms persisted after broad-spectrum antibiotics. Employees diagnosed with TB outside the project were asked to report to their project nurse and provide three sputum specimens for smear and culture, with the incentive of household screening. We monitored employer TB notifications, routinely sent by the Environmental Health Department of Harare City Health to notify employers of TB if a workplace address is provided by the patient on registration, and screened workers retiring because of ill health, or off sick for more than 2 wk.

### HIV Care

Workers testing HIV positive by VCT were provided with basic HIV care including cotrimoxazole (if in WHO stages 2 to 4) and isoniazid preventive therapy for 6 mo (if the TST ≥ 5 mm, or if previously treated for TB > 2 y earlier) [11]. Active TB was excluded (symptom screening, with chest radiography and TB smears and cultures if symptomatic) immediately before starting isoniazid. (HIV-positive employees were not otherwise routinely screened for tuberculosis.) Antiretroviral therapy (ART) was not widely available in Zimbabwe at the time of this study and was not provided, although referral was made to outside providers.

### Prevalence Survey

After 2 y, each workforce was screened for TB using symptoms (cough, fever, hemoptysis, night sweats, and unintentional weight loss) and TB cultures. Three sputum specimens were collected from all participants. Smears and cultures were processed immediately for participants with one or more symptoms. For asymptomatic participants, a single pooled specimen was cultured, with storage of three smears for reading if the pooled culture was positive. Concentrates were stored at −20 °C, and retrieved for redecontamination and repeat culture in case of initial culture contamination. Confidential HIV tests were requested, with the option of VCT. TB suspects (symptoms or positive screening culture) had repeat sputum microscopy, culture, and chest radiography, and were followed until TB was confirmed or excluded.

### TB Case Definitions

#### Incident TB.

Definite TB was defined as compatible illness plus positive smear or growth of M. tuberculosis from two or more sputum specimens, or positive smear or growth of M. tuberculosis from one specimen with suggestive radiological abnormalities and response to TB treatment.

Probable TB was defined as compatible illness with response to TB treatment following failure to respond to broad-spectrum antibiotics. Suspected pulmonary, pleural, or miliary TB was confirmed by radiological response. Radiographs were read by two independent readers. Other forms of extrapulmonary TB were confirmed by clinical response (weight gain and resolution of symptoms within 2 mo).

A number of patients were diagnosed and started on TB treatment by outside providers. TB clinic records, sputum smear results, and radiographs were used to apply case definitions, but cultures were not usually obtained before treatment was started. For the purposes of analysis, we have assumed that such patients were culture positive if smear positive, and culture negative if smear negative.

#### Prevalent TB.

Case definitions could not be met on the basis of prevalence screen results alone: additional evidence of TB from follow-up investigations was required, such as confirmatory positive smears or cultures, or radiological evidence of TB. Patients taking TB treatment were not considered to have active TB unless they were still culture positive. Screening (not follow-up) results defined smear and culture status for the point prevalence estimates.

Definite TB was defined as growth of M. tuberculosis from two or more sputum specimens, or from one specimen together with suggestive radiological abnormalities.

Probable TB was defined as compatible radiological features, with response to TB treatment within 2 mo following failure to respond to broad-spectrum antibiotics.

### Laboratory Methods

Decontamination with 4% NaOH and Lowenstein-Jensen (LJ) slopes were used for TB culture. M. tuberculosis was speciated by colonial morphology and failure to grow at room temperature and at 45 °C, and on PNB-containing LJ slopes. Both negative and positive controls were routinely included in each batch of cultures. Concentrated smears were stained with auramine O and read by fluorescence microscopy by two readers. Only smears subsequently confirmed with Ziehl-Neelsen staining were reported as positive.

Confidential HIV serology used Determine (Abbott Diagnostics, http://www.abbottdiagnostics.com), with all positives and 10% of negative specimens being confirmed by Unigold (Trinity Biotech, http://www.trinitybiotech.com) for serum specimens. Dried blood spots or oral mucosal transudate were collected from participants not willing to provide serum, and were tested using Determine/Vironostika and OraQuick/Vironostika (OraQuick, Abbott Diagnostics; Vironostika, bioMérieux, http://www.biomerieux.com), respectively.

### Ethical Approval

Approval was granted by the Ethics Committees of the London School of Hygiene and Tropical Medicine, Biomedical Research and Training Institute, Harare, Zimbabwe, and Medical Research Council of Zimbabwe, Harare, Zimbabwe. Written informed consent was provided by all participants. All information obtained from participants was kept confidential. Individual medical records were stored in locked cupboards at the respective company clinics. At the end of the study period, medical records were made available to remaining clinic staff, if shared confidentiality was requested by the employee, to enable ongoing care; otherwise medical records were removed.

### Data Analysis

Data were captured using EpiInfo 2002 (Centers for Disease Control and Prevention, http://www.cdc.gov/EpiInfo) and analyzed with Stata 7.0 software (http://www.stata.com).

Follow-up of each workforce started when TB diagnosis became available at company clinics and continued for 2 y. Follow-up for any given employee was from the later of either the date that workforce follow-up started or the date of joining the workforce, until the earlier of either the date that workforce follow-up stopped or the date of leaving the workforce. The prevalence survey started the day after follow-up finished at each site, and so prevalent cases were not included in the incident cohort analysis. Sixty-one participants were HIV negative at baseline, but HIV positive at the prevalence survey. None had incident or prevalent TB. Their data are analyzed as HIV negative for the incidence cohort and HIV positive for the prevalence survey.

Poisson regression and logistic regression were used for multivariate analysis of the cohort and cross-sectional data, respectively. Adjusted odds and rate ratios were used to calculate adjusted population-attributable fractions (PAFs) [12]. Robust confidence intervals (CIs) were used to adjust for clustering by site. Bootstrapping was used to calculate 95% confidence limits for disease duration estimates based on the ratio of prevalence and incidence of TB, and for disease duration ratios [5].

## Results

**Figure 1:**
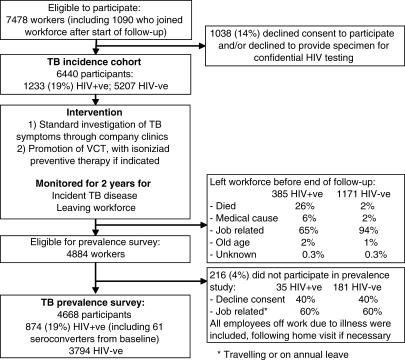
Study Profile HIV-ve, HIV negative; HIV+ve, HIV positive.

Participation rates are shown in Figure 1. Baseline characteristics of the 6,440 participants are shown by HIV status in Table 1. HIV prevalence was 19%. HIV-positive workers were more likely to have been treated for TB in the past (11% versus 2%, *p* = 0.027), to have had household contact with a TB patient (22% versus 15%, *p* < 0.001), and to be middle aged (*p* = 0.01). They were also more likely to be current or former smokers and to be manual workers (*p* < 0.001 for both). Workforce turnover was higher for HIV-positive than HIV-negative workers, with 69% and 78%, respectively, remaining in the workforce at the end of follow-up (*p* < 0.001).

**Table 1:**
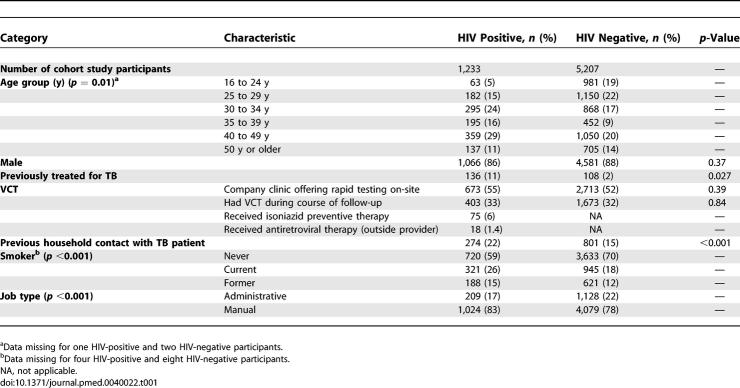
Baseline Characteristics

### Incidence of TB Disease

**Figure 2:**
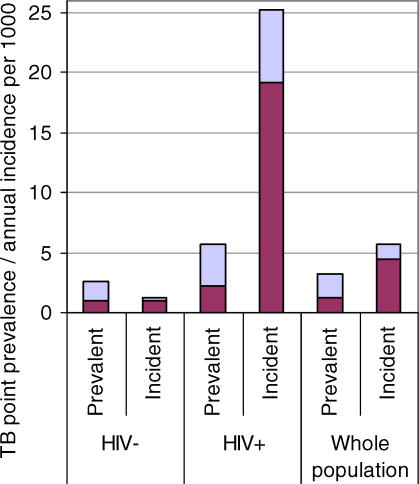
Point Prevalence and Annual Incidence Rates for Smear-Positive and Culture-Positive TB, According to HIV Status Columns are divided into smear-positive (dark portion) and smear-negative culture-positive TB (light portion).

A total of 106 patients with definite or probable TB occurred during cohort follow-up, of whom 61 (58%) were smear or culture positive. An additional 11 patients were treated for TB that did not meet case definitions. Overall TB incidence was 9.9 (95% CI = 7.8 to 12.9) per 1,000 person-years follow-up for all definite and probable disease. A breakdown of incidence rates by HIV status and smear and culture category is shown in Table 2 and Figure 2. Univariate- and multivariate-adjusted incidence rate ratios (IRRs) and PAFs for all TB cases and for culture-positive TB disease are shown in Table 3.

**Table 2:**
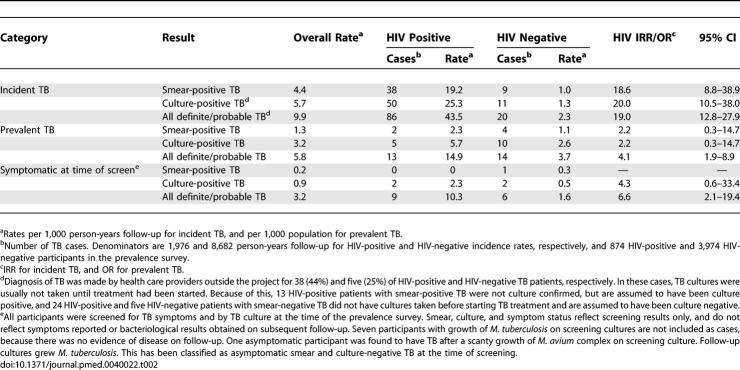
TB Incidence Rates (per 100,000 Person-Years Follow-Up), TB Point Prevalence per 100,000 Population, and HIV Rate Ratios for Incident and Prevalent TB

**Table 3:**
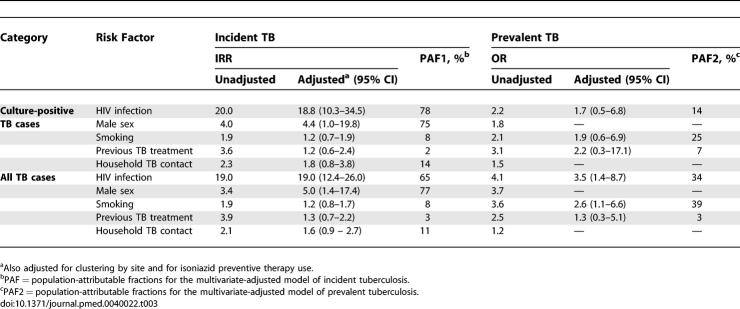
Risk Factors for Incident and Prevalent TB: Multivariate-Adjusted IRRs and ORs, and Population Attributable Fractions

#### Incidence and risk factors for culture-positive TB disease.

The incidence of culture-positive TB was significantly higher in HIV-positive than HIV-negative participants (overall rates, 25.3 and 1.3 per 1,000 person-years follow-up, respectively; univariate IRR = 20.0 (95% CI = 10.5 to 38.0). Previous TB treatment (IRR = 3.6; 95% CI = 1.9 to 6.9), male sex (IRR = 4.0; 95% CI = 0.9 to 17.8), being a current or former smoker (IRR = 1.9; 95% CI = 1.2 to 3.0), older age (IRR = 1.2 per group above 16 to 24 y; 95% CI = 1.1 to 1.3), manual job type (IRR = 1.3; 95% CI = 1.04 to 1.6), and having been a household contact of a TB patient (IRR = 2.3; 95% CI = 1.1 to 5.0) were each significantly associated with an increased rate of culture-positive TB.

However, there was considerable confounding by HIV, and on multivariate analysis, only HIV (adjusted IRR = 18.8; 95% CI = 10.3 to 34.5) and male sex (adjusted IRR = 4.4; CI = 1.0 to 19.8) remained significant (Table 3). Adjusted PAFs are also shown in Table 3, with the highest fractions (78% and 75%, respectively) being for HIV infection and male sex.

### Prevalent TB Disease

A total of 4,668 cohort participants (874 HIV positive and 3,794 HIV negative) were screened for prevalent TB at the end of follow-up. The participation rate for employees remaining in employment was 96%. Active TB was detected in 27 participants, giving a workforce point prevalence of 5.8 per 1,000 for all TB, and 3.2 and 1.3 per 1,000 for culture-positive and smear-positive TB, respectively.

A breakdown of prevalence by HIV status and smear and culture category is shown in Table 2 and Figure 2. A high proportion of patients with prevalent TB had subclinical disease at the time of screening, with 11 (41% of all prevalent cases and 73% of culture-positive cases) detected only through screening culture. Symptoms had developed in all but two by follow-up.

Univariate- and multivariate-adjusted odds ratios (ORs) are shown in Table 3, together with PAFs for variables included in the multivariate models. HIV infection was a significant risk factor for prevalent TB (unadjusted OR = 4.1; 95% CI = 1.9 to 8.9), as were smoking (unadjusted OR = 3.6; 95% CI = 1.5 to 8.8), and older age (unadjusted OR = 1.2 per category above 16 to 24 y; 95% CI = 1.05 to 1.4). Previous TB treatment was not a significant risk factor for prevalent TB in either univariate or multivariate analysis (PAF = 3% in both analyses).

#### Risk factors for prevalent culture-positive TB disease.

Statistical power was limited by the small numbers, but there were no significant risk factors for prevalent culture-positive TB. The adjusted odds ratio and PAF for HIV infection were 1.7 (95% CI = 0.5 to 6.8) and 14%, respectively.

#### Duration of disease before TB diagnosis.

The ratio of prevalent to incident TB provides an estimate of mean duration of disease before diagnosis (a measure of the case-detection rate) [5]. Precision was limited by small numbers, but duration was significantly shorter for HIV-related TB, with mean duration of smear positivity of 6 wk, and of 12 wk for culture positivity. This compares with 53 and 108 wk, respectively, for HIV-negative TB. The ratio of duration for HIV-positive compared to HIV-negative TB was 0.12 (95% CI = 0 to 0.70) for smear positivity and 0.11 (95% CI = 0.02 to 0.37) for culture positivity (Table 4).

**Table 4:**
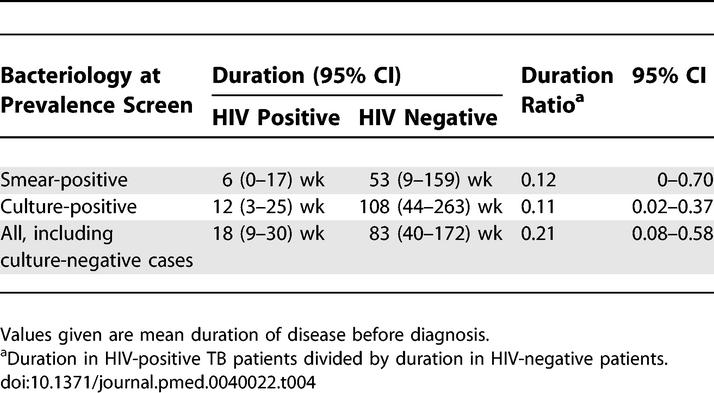
Indirect (Point Prevalence Divided by Incidence) Estimates of Duration of Infectiousness before Diagnosis of TB Disease, According to HIV Status

### Effect of VCT, Isoniazid Preventive Therapy, and HIV Care on TB Epidemiology

In addition to facilitated diagnosis, employees were provided with VCT linked to isoniazid preventive therapy. A total of 403 (33%) HIV-positive workers had VCT during the course of the study, and so became eligible for HIV care, and 75 (6% of all HIV-infected employees) received isoniazid. The main factor limiting isoniazid delivery was a low rate of tuberculin positivity (25% of TST-tested HIV-positive participants), as reported elsewhere in Africa [13–15]. TB was diagnosed in eight employees (9% of all incident TB cases in HIV-positive employees) as the result of preisoniazid screening. The potential impact of the HIV care program on incident and prevalent TB was, therefore, limited (employees were not screened for active TB unless they were TST positive or otherwise eligible for isoniazid) and is not considered here because of the statistical complexities of including an intervention delivered through a cluster-randomised trial design. Similarly, antiretroviral therapy is known to affect TB incidence [16], but was only accessed by 21 employees during this study, and so is not considered in the analysis.

## Discussion

**Figure 3:**
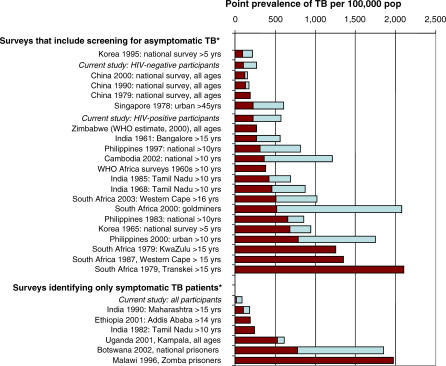
Adult and Whole Population Estimates of the Point Prevalence of Infectious TB Disease (per 100,000 Population) in Africa and Asia Bars are divided into smear-positive (dark portion) and smear-negative culture-positive TB (light portion). Not all studies used cultures, so only the prevalence of smear-positive disease is shown for these. Italic text indicates the current study results (HIV-positive participants, HIV-negative participants, and overall symptomatic burden). Otherwise, data were extracted from references [5,17–25] (Africa) and [26–34] (Asia). Ranking is by point prevalence of smear-positive disease. Whole-population estimates are typically 30%–50% lower than adult point prevalence estimates, because point prevalence of TB is negligible in children. Adult prevalence is shown whenever possible. * Methodology varied, but studies in which asymptomatic participants were screened for disease are included in the top half of the graph, whereas the bottom half shows results from studies in which only participants reporting one or more TB symptoms were screened further, with comparable results for symptomatic TB from the current study.

The results of this study suggest that passive case finding and treatment, the cornerstone of global TB control, may still be an effective way to control prevalent TB in high HIV prevalence populations, even when control of TB incidence has been apparently unsuccessful. This has major implications for TB control prospects, as it supports an approach whereby DOTS retains the essential role of controlling TB transmission rates, with the addition of integrated HIV/TB care aimed at providing individual protection from the very high risks of TB morbidity and mortality that are a striking feature of HIV disease in TB endemic areas [4]. The overall point prevalence of smear-positive TB after 2 y of easy access to diagnosis was 1.3 per 1,000 population in this study. This is considerably lower than most other reports of adult and whole-population point prevalence estimates from Africa [5,17–25] and Asia [26–34], and below the burden in Africa [17] in the pre-TB treatment era (Figure 3). Although this may in part reflect a “healthy worker” effect in this study, the high HIV prevalence of 19%, our inclusion of all employees who were on sick-leave during the prevalence survey, and the high TB incidence rates argue against this as the sole effect. Of note, our study population was predominantly male and middle-aged, both of which are strong risk factors for prevalent TB disease in other settings [26–29].

A low point prevalence of infectious TB is likely to be associated with low TB transmission rates. In this respect, the very low point prevalence of symptomatic infectious TB (0.9 per 1,000 population for culture-positive TB) is also notable. Symptomatic TB disease developed within a few weeks in all but two of the initially asymptomatic patients detected through the prevalence survey. Patients with subclinical TB have been consistently reported from previous prevalence surveys [27,30,35,36], and present a particular challenge to delivery of preventive therapy because they are at high risk of being inadvertently considered disease-free and so started on preventive therapy, usually a single drug. The high proportion of subclinical cases in this prevalence survey may reflect the unusually good access to investigation of TB symptoms with sensitive smears and culture. Infectiousness may be low in relatively asymptomatic patients, since coughing is important in TB transmission, and so control of secondary TB transmission events may be even better than is implied by the overall point prevalence estimates.

The results of this study are consistent with our previous finding in South African gold miners that HIV has a less pronounced impact on the point prevalence of active TB disease than on TB incidence rates, at least in the context of ready access to diagnosis of symptomatic TB [5]. The standard finding among HIV-negative persons that the burden of prevalent TB disease exceeds the annual TB incidence rate was reversed among our HIV-positive participants (Figure 2). This may be an intrinsic feature of HIV-related TB, due to rapid progression of TB disease and early onset of symptoms resulting in more rapid case finding as the duration of disease before diagnosis was significantly shorter for HIV-positive than HIV-negative TB patients in this and in the South African study [5]. Future studies are now needed to establish whether this relationship holds when access to diagnosis is more limited, and whether it can be replicated without use of culture and sensitive microscopy as first-line investigations.

As in South Africa [5], most patients in this study with prevalent smear- or culture-positive TB were HIV negative, and overall point prevalence predominantly reflected control in the HIV-negative subpopulation. In the current study, only 33% of patients with prevalent culture-positive TB were HIV positive compared to 82% of those with incident culture-positive TB, and the adjusted PAFs for HIV were much lower for prevalent than incident culture-positive TB (14% and 78%, respectively). Prevalent smear-positive TB is the primary driving force of TB transmission, and the finding that the impact of HIV on prevalent smear-positive TB is relatively modest is consistent with the finding that DOTS appears to have been more successful in controlling TB transmission than TB incidence rates in some high HIV prevalence countries [37–39].

The HIV diagnosis and care provided during the study may have contributed to rapid diagnosis of TB. Once diagnosed, HIV-positive workers were offered regular 3-monthly follow-up, and were treated for latent TB infection if they were tuberculin positive. However, only 23% of HIV-positive TB patients had VCT before their TB diagnosis, and TB incidence rates remained high despite the intervention. This was anticipated [40]. We have not considered the effects of isoniazid preventive therapy, administered during the course of the study, or antiretroviral therapy, because of the low coverage and complexity of adjusting for these effects. One other limitation is that capture of incident TB cases may have been incomplete, so that TB incidence may have been underestimated.

The power to identify risk factors for prevalent TB was limited by the success of the intervention, leading to low point prevalence, but HIV infection and smoking were of borderline significance on multivariate analysis when all TB cases (including culture negative) were included. Smoking has been identified as an important cause of morbidity and mortality from TB in India [41], but in this study the effect of smoking was less marked and was nonsignificant once adjustment for confounding with HIV status was made. Previous TB treatment, a major risk factor for prevalent TB disease in the pre-DOTS era [42], was not a significant risk factor for prevalent TB disease in this population, although we relied on self-reporting and so may have had incomplete capture.

In summary, we have demonstrated that a strong program based on case finding and treatment of self-presenting TB patients was associated with a burden of prevalent infectious TB disease well below annual TB incidence rates in this high HIV prevalence population. Most patients with prevalent smear- or culture-positive TB were HIV negative and asymptomatic at the time of screening, as previously reported from a similar study in South Africa [5]. This underscores the importance of including HIV-negative individuals in intensified TB control efforts. There has been a growing consensus that efforts to control TB in Africa need to be intensified [4,43,44]. The new global TB control strategy, the Global Plan to Stop TB 2006–2015, emphasises the need to apply existing diagnostic techniques, such as culture and sensitive microscopy techniques as used in this study, as well as the need to develop new diagnostic tools and expand joint TB/HIV activities [43]. Mathematical modelling suggests that better case finding and treatment for infectious TB will be among the most effective and cost-effective interventions [45,46]. Given the already brief duration of HIV-related TB disease before self-presentation, the added value of routine periodic screening for asymptomatic TB among known HIV-positive persons needs to be determined [4,43]. It will also be important to investigate the impact of antiretroviral therapy on the subclinical period of infectiousness among HIV-positive TB patients [4]. If generalisable, the current study results imply that well-implemented, intensified case finding and treatment may prove capable of reducing TB incidence in high HIV prevalence settings in the long term, despite the disappointing short-term response [4,44], if applied widely and intensively enough to maintain low levels of prevalent infectious TB for a sufficiently prolonged period.

## Abbreviations

CI: confidence interval
DOTS: directly observed treatment short course
IRR: incidence rate ratios
OR: odds ratio
PAF: population-attributable fraction
TB: tuberculosis
TST: tuberculin skin test
VCT: voluntary counselling and HIV testing
WHO: World Health Organization
